# Dietary Antioxidant Curcumin Mitigates CuO Nanoparticle-Induced Cytotoxicity through the Oxidative Stress Pathway in Human Placental Cells

**DOI:** 10.3390/molecules27217378

**Published:** 2022-10-30

**Authors:** Maqusood Ahamed, Rashid Lateef, Mohd Javed Akhtar, Pavan Rajanahalli

**Affiliations:** 1 King Abdullah Institute for Nanotechnology, King Saud University, Riyadh 11451, Saudi Arabia; 2 Department of Biochemistry, Faculty of Science, Veer Bahadur Singh Purvanchal University, Jaunpur 222003, Uttar Pradesh, India; 3 Department of Biology, University of Tampa, Tampa, FL 33569, USA

**Keywords:** CuO NPs exposure, pregnancy complications, BeWo cells, toxicity, apoptosis, prevention, turmeric

## Abstract

The placenta is an important organ that maintains a healthy pregnancy by transporting nutrients to the fetus and removing waste from the fetus. It also acts as a barrier to protect the fetus from hazardous materials. Recent studies have indicated that nanoparticles (NPs) can cross the placental barrier and pose a health risk to the developing fetus. The high production and widespread application of copper oxide (CuO) NPs may lead to higher exposure to humans, raising concerns of health hazards, especially in vulnerable life stages, e.g., pregnancy. Oxidative stress plays a crucial role in the pathogenesis of adverse pregnancy outcomes. Due to its strong antioxidant activity, dietary curcumin can act as a therapeutic agent for adverse pregnancy. There is limited knowledge on the hazardous effects of CuO NPs during pregnancy and their mitigation by curcumin. This study aimed to investigate the preventive effect of curcumin against CuO NP-induced toxicity in human placental (BeWo) cells. CuO NPs were synthesized by a facile hydrothermal process and characterized by X-ray diffraction, scanning electron microscopy, transmission electron microscopy, and photoluminescence techniques. We observed that curcumin did not induce toxicity in BeWo cells (1–100 µg/mL for 24 h), whereas CuO NPs decreased the cell viability dose-dependently (5–200 µg/mL for 24 h). Interestingly, CuO NP-induced cytotoxicity was effectively mitigated by curcumin co-exposure. The apoptosis data also exhibited that CuO NPs modulate the expression of several genes (p53, bax, bcl-2, casp3, and casp9), the activity of enzymes (caspase-3 and -9), and mitochondrial membrane potential loss, which was successfully reverted by co-treatment with curcumin. The mechanistic study suggested that CuO-induced reactive oxygen species generation, lipid peroxidation, and higher levels of hydrogen peroxide were significantly alleviated by curcumin co-exposure. Moreover, glutathione depletion and the lower activity of antioxidant enzymes (superoxide dismutase, glutathione peroxidase, and catalase) were effectively mitigated by curcumin. We believe this is the first report exhibiting that CuO-induced toxicity in BeWo cells can be effectively alleviated by curcumin. The pharmacological potential of dietary curcumin in NP-induced toxicity during pregnancy warrants further investigation.

## 1. Introduction

Copper oxide nanoparticles (CuO NPs) have attracted the attention of researchers for their exceptional optical, electrical, and electromagnetic properties [1]. CuO NPs are increasingly utilized in several fields such as catalysis, gas sensors, batteries, solar cells, paint, the food industry, and textiles [2,3]. Due to their inherent antimicrobial activity, CuO NPs have shown the potential to be utilized in various household and medical products [4]. A recent study estimated that from the year 2020–2025, the global consumption of CuO NPs will be around 200–800 tons each year [5]. The high production and widespread application of CuO NPs may lead to a higher exposure to the human body, raising the concern of human health hazards, especially in vulnerable life stages (e.g., pregnancy) that can affect intrauterine fetal growth [6]. Pregnancy complications generate multiple diseases with detrimental effects on maternal and fetal health.

The placenta is a key organ in maintaining a healthy pregnancy. The placenta is involved in various functions including the transport of O_2_ and nutrients to the fetus, and the removal of CO_2_ and waste materials from the fetus [7]. The predecessor cells of the placenta are the trophoblasts that have the potential to differentiate into all other cell types of the placenta such as syncytiotrophoblasts and cytotrophoblasts. These cells play an indispensable role in maintaining a dynamic interaction between the mother and fetus. These cells can be considered as the first fetal derived cells that encounter any compounds present in the maternal blood. In addition, the placenta expresses a number of xenobiotic transporters and acts as a barrier in protecting the fetus from harmful drugs and environmental pollutants [8]. Impaired placental function has been associated with several pregnancy complications, e.g., preeclampsia, gestational diabetes, premature delivery, and specific birth defects [7]. 

Exposure to nanomaterials may interfere with the development and function of the placenta such that it affects the development of the fetus [9]. There is a growing field of evidence that nanomaterials can cross the placental barrier in pregnant mice and induce toxicity in mothers and developing fetuses [10,11]. A recent study observed that maternal pulmonary exposure to nanopolystyrene leads to the translocation of particles to placental and fetal tissues and renders the fetoplacental entity susceptible to hazardous effects [12]. The oral exposure of pregnant mice to >180 mg/kg ZnO NPs at gestation day 10.5 to 17.5 might induce maternal injury, fetal growth restriction, and a reduction in fetal number. Moreover, a treatment of 540 mg/kg ZnO NPs could induce elemental Zn to cross the placental barrier [13]. Hong et al. [14] found that maternal exposure (gestational day 0–17) to 100 mg/kg TiO_2_ NPs distinctly decreased maternal weight gain, placental weight, fetal weight, the number of live fetuses, and the rate of development of the fetal skeleton. Hence, it is imperative to study the placental toxicity of widely used metal oxide NPs (e.g., CuO NPs) in order to understand the possible mechanisms for mitigating their toxicity during pregnancy. Curcumin is a polyphenolic compound found in the rhizome of turmeric (*Curcuma longa* L.), a plant of the Zingiberaceae family. Curcumin shows several health benefits and has been utilized since ancient times for both food and pharmaceutical reasons [15]. A recent review stated that the oral use of turmeric and curcumin did not result in reproductive toxicity in animals at certain doses. Studies on humans similarly showed no toxic effects, and curcumin was found to be safe at the dose of 6 g/day orally for 4–7 weeks [16]. Curcumin possesses a number of properties including antioxidant, anticancer, anti-inflammatory, and anti-toxicant; hence, this compound can act as a therapeutic agent to counter several health complications including adverse pregnancy outcomes [17]. In vitro and in vivo studies have shown that curcumin positively regulates key pathophysiological pathways involved in the most common pregnancy-related complications including intrauterine growth restriction (IUGR), preeclampsia, gestational diabetes, and preterm delivery [18]. For instance, the daily administration of curcumin (400 mg/kg) to IUGR mice may be useful in preventing IUGR-induced oxidative stress and inflammation, and eventually reducing the occurrence of IUGR [19]. Oxidative stress, apoptosis, and inflammation are common conditions normally found in almost all of the pathological placental conditions [18]. There is strong evidence that CuO NPs induce toxicity in biological systems through oxidative stress and apoptosis [20,21,22]. Due to its strong antioxidant activity, curcumin can play a key role in improving the pregnancy complications caused by CuO NP exposure. Information on the hazardous effects of CuO NPs during pregnancy and their mitigation by dietary curcumin is scarce.

This study was designed to investigate the preventive effect of curcumin against CuO NP-induced cytotoxicity and apoptosis in human placental (BeWo) cells. The potential mechanism of the mitigating effect of curcumin against CuO NP-induced toxicity was delineated through the oxidative stress pathway. The BeWo cell line originates from human placental choriocarcinoma epithelial cells and has been widely used as a model to investigate the placental toxicity of drugs and xenobiotics [23,24].

## 2. Materials and Methods

### 2.1. Materials and Regents

Copper nitrate (CuNO_3_.2H_2_O), MTT, neutral red probe, propidium iodide (PI) NaOH, tetramethylrhodamine methyl ester (TMRM), 2′-7′-dichlorodihydrofluorescein diacetate (H_2_DCFDA), glutathione (GSH), and Ellman reagents were purchased from Sigma-Aldrich, St. Louis, MO, USA. Dulbecco’s Modified Eagle Medium (DMEM), fetal bovine serum (FBS), glutamine, streptomycin–penicillin, and trypsin were obtained from Carlsbad, CA, USA. 

### 2.2. Synthesis of CuO NPs

CuO NPs were synthesized by a facile hydrothermal process utilizing copper nitrate (CuNO_3_·2H_2_O) as a precursor. Briefly, 0.1 M copper nitrate was dissolved in 100 mL of de-ionized water. Further, 0.4 M NaOH solution was added into the copper nitrate solution drop-wise with constant stirring (magnetic stirring 500 rpm at room temperature) to reach pH 11. Then, the reaction mixture was stirred at 60 °C until the light-colored mixture turned into black. The reaction mixture was further transferred into a Teflon-coated autoclave and heated at 120 °C for 6 h and allowed to cool at room temperature. The reaction mixture was filtered through Wattman no. 1 fine filter paper and the filtrate was washed 3 times with water and ethanol. The filtrate product was dried in a vacuum oven at 100 °C for 6 h. The obtained powder was finally calcined at 600 °C for 4 h.

### 2.3. Characterization of CuO NPs

The crystallinity and phase purity of the prepared CuO NPs were examined by X-ray diffraction (XRD) (PanAnalytic X`Pert Pro) (Malvern Instruments, WR14 1XZ, UK) using Cu-Kα radiation (λ = 0.154 nm at 45 kV and 40 mA). Photoluminescence spectra of CuO NPs were assessed using a fluorescence spectrometer (DW-F97 Drawell, Shanghai, China). Structural characterization (e.g., morphology, shape, and size) was further carried out by field-emission scanning electron microscopy (FESEM, JSM-7600F, JEOL, Inc., Tokyo, Japan) and field-emission transmission electron microscopy (FETEM, JEM-2100F, JEOL). The elemental composition of the prepared CuO NPs was examined by energy-dispersive X-ray spectroscopy (EDS).

### 2.4. Cell Culture and Exposure Protocol

A human placental cell line (BeWo) was obtained from the American Type Cell Culture Collection (ATCC, Manassas, VA, USA) and cultured in Dulbecco’s Modified Eagle Medium (DMEM, Carlsbad, CA, USA) supplemented with 10% fetal bovine serum, 2 mM glutamine, 100 µg/mL streptomycin, and 100 unit/mL penicillin. Cells were used up to 5 passages. Cells were maintained at 37 °C in a humidified incubator with a 5% CO_2_ supply. At 80–85% confluence, cells were harvested (0.25% trypsin) and sub-cultured for further experiments. Cells were seeded in plates and allowed for overnight to attach on the surface. Then, cells were exposed to different concentrations of CuO NPs and/or curcumin for 24 h. Cells without NPs or curcumin served as a negative control in each experiment. 

A stock suspension of CuO NPs (1 mg/mL) was prepared by dissolving in de-ionized water. A stock solution of curcumin was prepared by dissolving the compound in ethanol. Different concentrations of CuO NPs (1–200 µg/mL) were prepared by dilution in DMEM and sonication in a water bath sonicator for 10 min at 40 W to avoid agglomeration of the NPs before exposure to cells. Different concentrations of curcumin (1–200 µg/mL) were prepared by dilution of the stock solution of curcumin in DMEM and sonicated as described above.

### 2.5. Assay of Biochemical Parameters

An MTT cell viability assay was carried out as described earlier [25] with slight modifications [26]. The neutral red uptake (NRU) cell viability assay was carried out using the protocol of Borenfreund and Puerner [27]. The mRNA levels of apoptotic genes (p53, bax, bcl-2, casp3, and casp9) were estimated by real-time PCR (Applied Biosystems, Foster City, CA, USA), applying a SYBR green probe as described elsewhere [28]. The sequences of a specific set of primers for these genes including β-actin are given earlier [29]. The mRNA expression level of apoptotic genes was normalized with β-actin that was applied as an internal housekeeping control. The activity of caspase-3 and -9 enzymes was measured applying colorimetric kits of BioVision (Milpitas, CA, USA). Mitochondrial membrane potential (MMP) was examined by a microplate reader (Synergy-HT, BioTek, Winooski, VT, USA), as well as by a fluorescence microscope (DMi8, Leica Microsystems, GmbH, Wetzlar Germany) using the fluorescent probe tetramethylrhodamine methyl ester (TMRM) as reported previously [30] with minor changes [31]. The intracellular ROS level was assessed both quantitatively by a microplate reader (Synergy-HT, BioTek, CA, USA) and qualitatively by a fluorescence microscope (DMi8, Leica Microsystems) using 2′-7′-dichlorodihydrofluorescein diacetate (H_2_DCFDA) [32]. The cellular glutathione (GSH) level was quantified through Ellman’s method [33]. Malonaldehyde (MDA), an end product of lipid peroxidation, was examined using the protocol of Ohkawa [34]. A fluorometric assay of intracellular hydrogen peroxide (H_2_O_2_) level was carried out using a kit from Sigma-Aldrich. The activity of the superoxide dismutase (SOD) enzyme was assayed using a commercial kit (Cayman chemical, MI, USA). The activity of the glutathione peroxidase (GPx) enzyme was assayed applying the procedures of Rotruck et al. [35]. A colorimetric assay of catalase (CAT) enzyme activity was examined using the protocol of Sinha [36]. Bradford’s method was employed to estimate protein content [37]. The procedures of each biochemical assay are briefly described in the Supplementary Material.

### 2.6. Statistical Analysis

A one-way analysis of variance (ANOVA) followed by Dennett’s multiple comparison test was used to analyze the results. The threshold *p* < 0.05 was ascribed as significant. The quantitative data are presented as the mean ± SD of three independent experiments (*n* = 3).

## 3. Results and Discussion

### 3.1. Characterization Study

**Figure 1:**
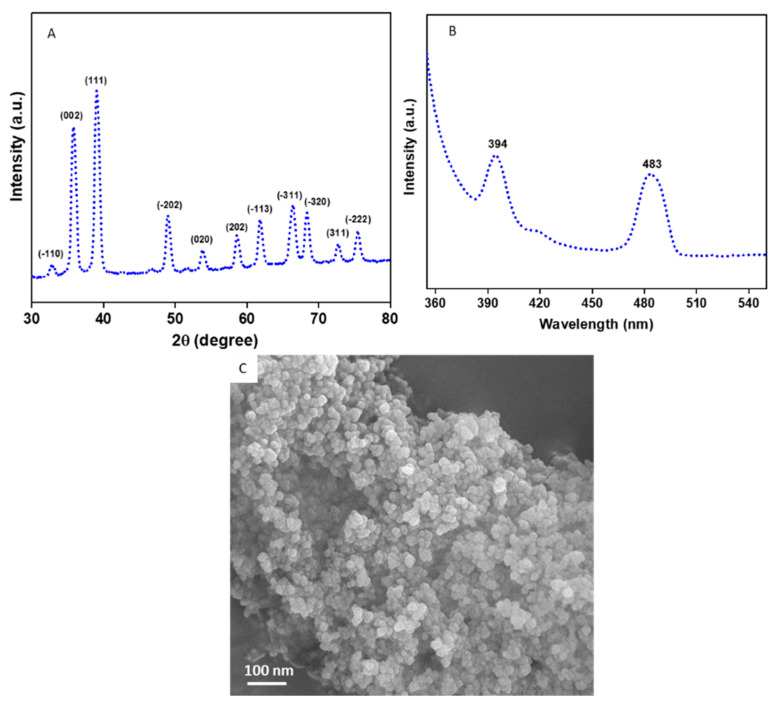
Characterization of CuO NPs. (**A**) XRD spectra, (**B**) PL spectra, and (**C**) FESEM micrograph. XRD: X-ray diffraction, PL: photoluminescence, FESEM: field-emission scanning electron microscopy.

Figure 1A provides the XRD spectra of the CuO NPs. All the diffraction peaks observed at 2θ were matched with JCPDS card no. 45-0937 which represents the monoclinic structure of CuO NPs [38,39]. The particle size of the CuO NPs was estimated from the most prominent peak (111) using Scherrer’s equation [40].

(1) $$d=\frac{K\lambda }{\beta \cos\theta }$$

where *d* is the size of the particle, *K* is known as Scherrer’s constant (*K* = 0.9), *λ* is the X-ray wavelength (1.54 Å), *θ* is the Bragg diffraction angle, and *β* is the full width at half maximum (FWHM). The average particle size of the CuO NPs was approximately 45 nm. Impurity peaks were not detected in XRD, which confirms the high purity of the prepared CuO NPs. Sharp and high intensity of peaks suggest the high crystalline nature of the CuO NPs. The photoluminescence (PL) spectra of the CuO NPs with a 320 nm excitation wavelength is presented in Figure 1B. Two prominent peaks were observed at 394 nm (violet) and 483 nm (blue) for the CuO NPs. The violet peak at 394 nm is related to band-edge emission due to electron-hole pair recombination in excitons. The blue peak at 483 is due to the electronic transition between oxygen interstitials and copper vacancies (from acceptor to donor energy levels) [3]. The FESEM micrograph demonstrates that the CuO NPs have smooth surfaces, spherical morphology, and some degree of agglomeration (Figure 1C).

**Figure 2:**
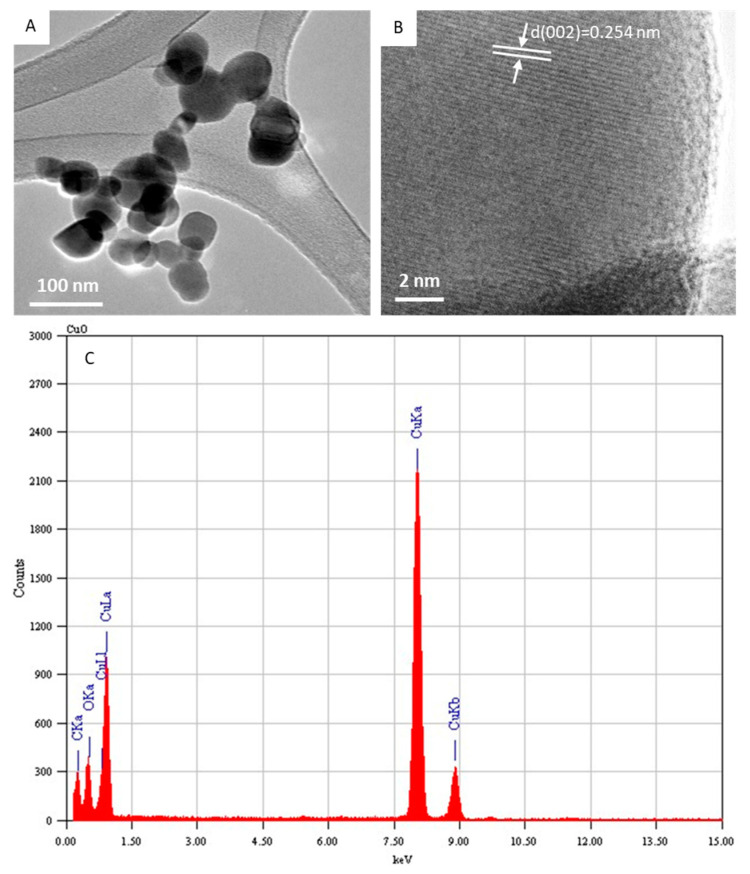
Characterization of CuO NPs. (**A**) Low-resolution FETEM micrograph, (**B**) high-resolution FETEM micrograph, and (**C**) EDS analysis. FETEM: field-emission transmission electron microscopy, EDS: energy-dispersive X-ray spectroscopy.

Figure 2A depicts the typical FETEM micrograph of the CuO NPs. This image demonstrated that the CuO NPs possess a spherical shape with some level of agglomeration. The average diameter was estimated from measuring over 100 particles in random fields of the FETEM micrograph view. The average particle diameter of the CuO NPs calculated from FETEM was around 43 nm, which was in agreement with the size calculated from XRD. In the high-resolution FETEM images (Figure 2B), the calculated interplanar distance was 0.254 nm, which corresponds to the (002) plane consistent with the monoclinic structure of the CuO phase. The TEM-associated EDS spectra of the CuO NPs indicated Cu and O peaks. The carbon peak was from the carbon-coated TEM grid. No other elemental impurities were detected in the EDS spectra (Figure 2C). The characterization data of the present CuO NPs were in accordance with recently published works [2,39].

### 3.2. Cytotoxicity Study

**Figure 3:**
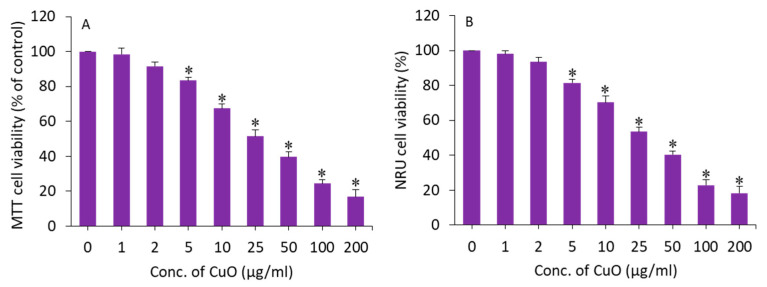
Dose-dependent cytotoxicity of CuO NPs in BeWo cells. (**A**) MTT assay. (**B**) NRU assay. * Statistically different from the controls (*p* < 0.05).

MTT and NRU assays were applied to assess the cytotoxic potential of CuO NPs in BeWo cells following exposure for 24 h to different concentrations (1–200 µg/mL) of this nanomaterial. An MTT assay measures the cell metabolic activity based on the ability of nicotinamide adenine dinucleotide phosphate (NADPH)-dependent cellular oxidoreductase enzymes to reduce a tetrazolium salt (MTT) into formazan crystals. The NRU assay is based on the ability of living cells to take up and bind neutral red (NR), a dye which easily penetrates cell membranes and accumulates in lysosomes. Both assays were conducted to confirm the cytotoxicity data. The exposure time (24 h) was selected based on our previous in vitro toxicity studies of CuO NPs [32,41]. In this study, both assays demonstrated that CuO NPs induce a dose-dependent cytotoxicity in BeWo cells. In the MTT results, cell viability was depleted to 98%, 92%, 83%, 67%, 51%, 40%, 24%, and 17% for the concentrations of 1, 2, 5, 10, 25, 50, 100, and 200 µg/mL, respectively (*p* < 0.05) (Figure 3A). The IC_50_ of the CuO NPs calculated from the MTT data was 23 µg/mL. Figure 3B displays the cytotoxicity data obtained from NRU assays. The NRU data were in accordance with the MTT results. The IC_50_ of the CuO NPs calculated from the NRU data was 25 µg/mL. The cytotoxicity data observed in this study were in accordance with other reports that have demonstrated the dose-dependent cytotoxicity of CuO NPs in different types of human cell lines such as lung cancer (A549) cells, normal lung (WI-38) cells, liver cancer (HepG2), and colorectal cancer (Caco-2) cells [32,42,43]. For example, CuO NPs in the concentration range of 2–50 µg/mL for 24 h induced dose-dependent cytotoxicity in HepG2 cells [32]. 

**Figure 4:**
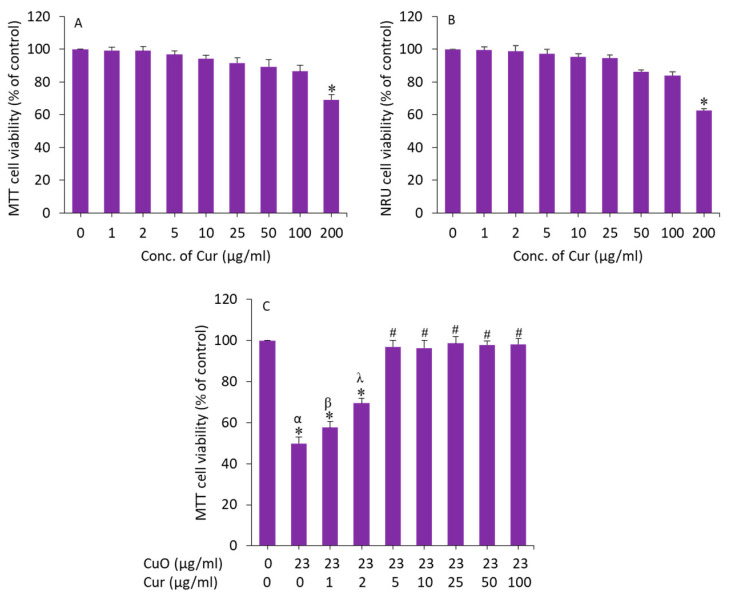
(**A**,**B**) Cytotoxicity of curcumin in BeWo cells. (**C**) Preventive effects of curcumin against CuO NP-induced cytotoxicity in BeWo cells. * Significantly different from the control group (*p* < 0.05). **#** Significantly different from the α, β, and λ groups (*p* < 0.05). Cur: curcumin.

We further examined the effect of curcumin on BeWo cells. Cells were exposed for 24 h to various concentrations (1, 2, 5, 10, 25, 50, 100, and 200 µg/mL) and cytotoxicity was examined by MTT assay. As we can see in Figure 4A, curcumin did not cause cytotoxicity to BeWo cells up to 100 µg/mL. Earlier studies have also observed the biocompatibility of curcumin in different types of mammalian cell lines such as human airway epithelial (HEp-2), human breast cancer (MCF-7), and embryonic rat heart-derived (H9c2) cells [44,45].

To determine the appropriate concentration of curcumin that effectively mitigates the toxicity of CuO NPs in BeWo cells, we prepared 23 µg/mL (IC_50_ value of MTT assay) of CuO NPs and co-exposed with different safe concentrations of curcumin (1–100 µg/mL) for 24 h, and the effect was examined by MTT assay. The results showed that curcumin at a dosage of 5 µg/mL achieved the maximum mitigating effect against CuO NPs (23 µg/mL) following induced cytotoxicity in BeWo cells (Figure 4B). Above the concentration of 5 µg/mL of curcumin, the alleviating effect against CuO NP-induced toxicity in BeWo cells was not statistically different. Based on these results, we selected a 5 µg/mL concentration of curcumin to explore the mechanism of its mitigating effect against CuO NPs (23 µg/mL, IC_50_ value) following induced toxicity in human placental BeWo cells. 

### 3.3. Apoptosis Study

**Figure 5:**
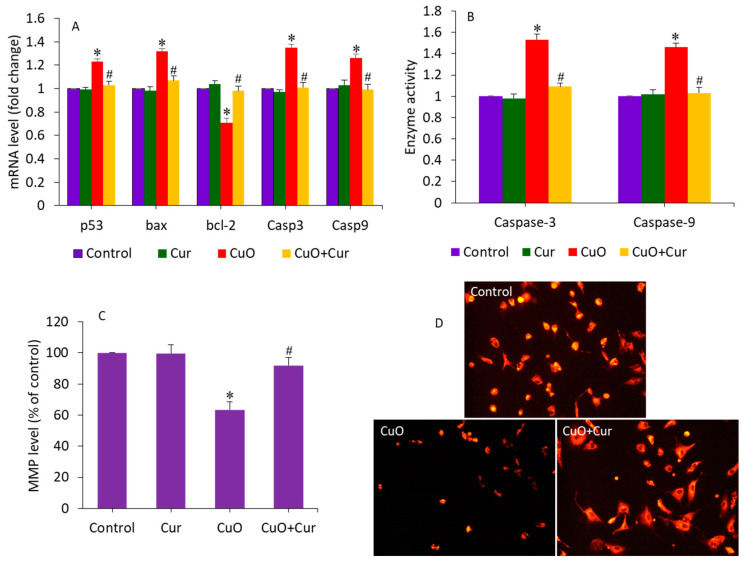
Apoptosis response in BeWo cells following exposure to CuO NPs (23 µg/mL) and/or curcumin (5 µg/mL) for 24 h. (**A**) The mRNA expression level of p53, bax, bcl-2, casp3, and casp9 genes. (**B**) Activity of caspase-3 and caspase-9 enzymes. (**C**) Quantitative analysis of MMP level. (**D**) Fluorescent microscopic analysis of MMP level. * Significantly different from the control group (*p* < 0.05). **#** Significantly different from the CuO NPs group (*p* < 0.05). Cur: curcumin.

Apoptosis, oxidative stress, and inflammation play indispensable roles in poor placental development and pose substantial risks to pregnancy [18,46]. Gestational exposure to various NPs (e.g., TiO_2_ and polystyrene) induces apoptosis in trophoblasts, which causes placental dysfunction and poor fetal development [46,47]. A recent study observed that maternal exposure to TiO_2_ NPs during pregnancy and lactation modifies the progeny’s hippocampal mRNA expression of Bax and bcl-2, induces apoptosis, and reduces neurogenesis [48]. Our earlier published data has also demonstrated that CuO NPs induce apoptosis in human liver HepG2 cells through the alteration of the expression of several apoptotic genes (p53, bax, bcl-2, casp3, and casp9) and MMP loss [32]. Moreover, curcumin was also found to alleviate methylglyoxal-induced apoptosis in mouse ESC-B5 cells and blastocysts, indicating its safety for the developing fetus. In this study, we examined the alleviating effect of dietary curcumin against CuO NP-induced apoptosis in BeWo cells by measuring the expression of several apoptosis genes (p53, bax, bcl-2, casp3, and casp9) and the MMP level. Cells were treated with CuO NPs (23 µg/mL) and/or curcumin (5 µg/mL) for 24 h. Figure 5A demonstrates that CuO NPs significantly altered the mRNA expression level of these apoptotic genes. The tumor suppression gene p53 and pro-apoptotic gene bcl-2 were significantly upregulated, while the anti-apoptotic gene bax was significantly downregulated following CuO NP exposure (*p* < 0.05). Since we observed a decrease in bcl-2 expression, we can hypothesize the role of this gene in CuO NP-induced apoptosis in placental cells. Bax insertion into the mitochondrial membrane can also trigger p53-mediated apoptotic cell death. Caspases are known to be activated during apoptosis in many cells and play important roles in both the initiation and execution of apoptosis. Moreover, the casp3 and casp9 genes were more highly regulated in cells treated with CuO NPs than in untreated control cells. Changes in the expression of these apoptotic genes upon exposure to CuO NPs have also been reported in previous studies [22,32]. Interestingly, co-exposure of curcumin significantly attenuated the effects of the CuO NP-induced dysregulation of these apoptotic genes (*p* < 0.05) (Figure 5A). In support of the mRNA expression data, we further examined the activity of the caspase-3 and -9 enzymes following exposure to CuO NPs and/or curcumin. Similar to the mRNA results, the CuO NP-induced higher activity of the caspase-3 and -9 enzymes were effectively mitigated by curcumin co-exposure (Figure 5B). Huang and co-workers observed that N-acetylcysteine (an FDA-approved antioxidant) prevents the multi-walled carbon nanotubes (MWCNTs), which induce DNA damage and fetal brain developmental abnormalities in pregnant mice [49].

Curcumin can prevent MMP loss, reducing the risk of premature rupture of the membrane—a key cause of preterm birth [19]. In this study, MMP loss, an indicator of early apoptosis, was also examined in BeWo cells following exposure to CuO NPs and/or curcumin for 24 h. Figure 5C shows that CuO NPs significantly decreased the MMP level in BeWo cells. Curcumin alone did not affect the MMP level of BeWo cells. Interestingly, in the co-exposure group (CuO + Cur), the MMP level significantly increased to a level almost similar to that of the control group (*p* < 0.05). MMP levels were also examined by fluorescent microscope (Figure 5D). These micrographs indicated that the fluorescent brightness of the TMRM probe was lower (an indicator of MMP loss) in CuO NP-treated cells. However, in the co-exposure group (CuO NPs + curcumin), the brightness of the TMRM probe almost rose to the level of the control. Altogether, CuO NP-induced apoptosis in BeWo cells was effectively mitigated by curcumin co-exposure. 

### 3.4. Oxidative Stress Study

**Figure 6:**
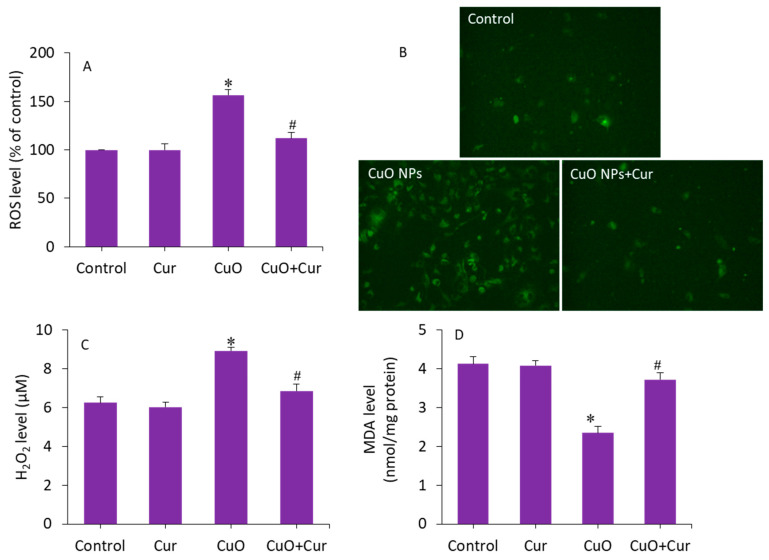
Pro-oxidant levels in BeWo cells following exposure to CuO NPs (23 µg/mL) and/or curcumin (5 µg/mL) for 24 h. (**A**) Quantitative analysis of ROS level. (**B**) Fluorescence microscopic analysis of ROS level. (**C**) H_2_O_2_ level. (**D**) MDA level. * Significantly different from the controls (*p* < 0.05). **#** Significant protective effect of curcumin against CuO NPs (*p* < 0.05). ROS: reactive oxygen species, H_2_O_2_: hydrogen peroxide, MDA: malondialdehyde, Cur: curcumin.

Excessive ROS production and oxidative stress in placental tissues due to NP exposure has been suggested as a potential mechanism for placental dysfunction and several pregnancy complications [50]. Therefore, antioxidant supplementation may protect placental trophoblasts from oxidative stress and lead to the mitigation of pregnancy disorders and reproductive toxicity [51]. To delineate the possible mechanisms of the protective effect of curcumin against CuO NP toxicity in BeWo cells, several pro-oxidant and antioxidant parameters were measured. Cells were treated with CuO NPs (23 µg/mL) and/or curcumin (5 µg/mL) for 24 h. Figure 6A demonstrates that CuO NP-induced intracellular ROS generation was significantly alleviated by curcumin co-exposure (*p* < 0.05). This quantitative analysis further supported the fluorescence microscopy study. The fluorescent micrographs depicted that the brightness of the dichlorofluorescein (DCF, an indicator of ROS generation) was increased in CuO NP-treated cells, and the co-exposure of curcumin remarkably reduced the ROS generation, as the brightness of the DCF probe decreased in the co-exposure (CuO NPs + curcumin) group (Figure 6B). Furthermore, CuO NP-induced H_2_O_2_ (a pro-oxidant) and MDA (an end product of lipid peroxidation) levels were effectively abrogated by curcumin co-exposure (*p* < 0.05) (Figure 6C,D). 

**Figure 7:**
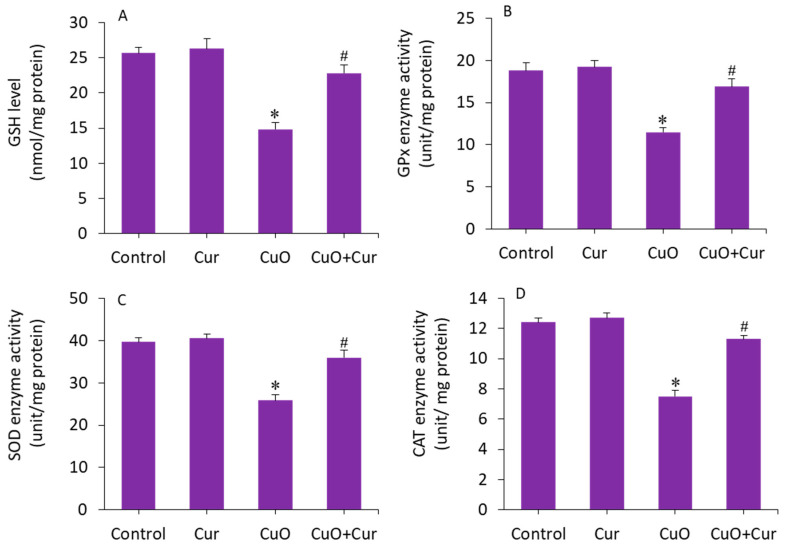
Antioxidant levels in BeWo cells following exposure to CuO NPs (23 µg/mL) and/or curcumin (5 µg/mL) for 24 h. (**A**) GSH level. (**B**) GPx activity. (**C**) SOD activity. (**D**) CAT activity. * Significantly different from the controls (*p* < 0.05). **#** Significant protective effect of curcumin against CuO NPs (*p* < 0.05). Cur: curcumin, GSH: glutathione, GPx: glutathione peroxidase, SOD: superoxide dismutase, CAT: catalase.

The beneficial role of curcumin in pregnancy is also due to its antioxidant capacity, reducing lipid peroxidation and maintaining the activity of various antioxidant enzymes [15]. The mitigating effect of curcumin against CuO NP-induced antioxidant depletion was further explored in BeWo cells. The results showed that GSH level and activity of antioxidant enzymes (GPx, SOD, and CAT) were lower in the CuO NP-exposed cells as compared with the control (*p* < 0.05). Interestingly, the CuO NP-induced depletion of antioxidant molecules (GSH) and enzymes (GPx, SOD, and CAT) in BeWo cells was efficiently restored by curcumin co-exposure (*p* < 0.05) (Figure 7A–D). Overall, we found that CuO NP-induced toxicity and apoptosis in BeWo cells was effectively alleviated by curcumin through oxidative stress and apoptosis.

## 4. Conclusions

In summary, the possible mechanism of the preventive effect of dietary curcumin against CuO NP toxicity was explored in human placental BeWo cells. We observed that CuO NP-induced cytotoxicity and apoptosis (altered regulation of p53, bax, bcl-2, casp3, and casp9 genes, and MMP loss) in BeWo cells were effectively attenuated by curcumin. Moreover, CuO NP-induced pro-oxidant generation (ROS, H_2_O_2_, and MDA) and antioxidant depletion (GSH, GPx, SOD, and CAT) were efficiently mitigated by curcumin, suggesting that the protective effects of curcumin are mediated through oxidative stress and apoptosis. The present work indicates that the dietary antioxidant curcumin could be a potential preventive agent against CuO NP-induced pregnancy complications. This study warrants further research on the attenuating effect of curcumin against metal oxide nanoparticle-induced pregnancy complications in suitable animal models. 

## Data Availability

The raw data will be available from corresponding author upon reasonable request.

## Supplementary Materials

The following are available online at https://www.mdpi.com/article/10.3390/molecules27217378/s1. The procedures of each biochemical assay are briefly described [25,29,32,33,34,35,36].
