# Supplementary material for: Dietary Antioxidant Curcumin Mitigates CuO Nanoparticle-Induced Cytotoxicity through the Oxidative Stress Pathway in Human Placental Cells
Source: Molecules. 2022 Oct 30;27(21):7378. doi: 10.3390/molecules27217378 (PMC9654626; doi:10.3390/molecules27217378)
Supplement: Supplementary file 1 [file molecules-27-07378-s001.zip › molecules-1980669-supplementary.pdf]

# Supplementary Materials

**Dietary antioxidant curcumin mitigates CuO nanoparticles induced cytotoxicity through oxidative stress pathway in human placental cells**

**Maqusood Ahamed[1] *, Rashid Lateef[2], Mohd Javed Akhtar[1] and Pavan Rajanahalli[3]**

[1] King Abdullah Institute for Nanotechnology, King Saud University, Riyadh-11451, Saudi Arabia

[2] Department of Biochemistry, Faculty of Science, Veer Bahadur Singh Purvanchal University, Jaupur-222003, Uttar Pradesh, India

[3] Department of Biology, University of Tampa, Tampa, Florida-33569, USA.

* Correspondence: mahamed@ksu.edu.sa (Maqusood Ahamed)

***MTT assay***

Cell viability was assessed by MTT assay (Mosmann, 1983) with some specific changes (Ahamed et al., 2011). Briefly, 20,000 cells/well seeded in a 96-well plate and allowed for 24 h to attach on the surface. Then, cells were treated for 24 h to different concentrations of CuO NPs (1-200 μg/ml) or curcumin (1-200 μg/ml). For some experiments, cells were also treated with CuO NPs and/or curcumin for 24 h. At the end of exposure time, culture medium was removed from each well to avoid interference of CuO NPs or curcumin and replaced with new medium containing MTT solution in an amount equal to 10% of culture volume. The 96-well plate is now incubated for 3 h at 37 °C until a purple colour formazan product was developed. The resulting formazan product was dissolved in acidified isopropanol. Further, 96-well plate was centrifuged at 2300×g for 5 min to settle down the debris, if present in the solution. Then, a 100 μl supernatant was transferred to other fresh wells of 96-well plate and absorbance was measured at 570 using a microplate reader (Synergy-HT, BioTek, Vinnoski, VT, USA).

***NRU assay***

Neutral red uptake (NRU) assay was performed following the procedure as described by Borenfreund and Puerner (1984) with some modifications (Ahamed et al., 2011). In brief, 20, 000 cells/well were seeded in 96-well plates and exposed to different concentrations of CuO NPs (1-200 μg/ml) or curcumin (1-200 μg/ml). for 24 h. For some experiments, cells were also treated with CuO NPs and/or curcumin for 24 h. At the end of the exposure time, test solution was aspirated and cells were washed with phosphate buffer saline (PBS) twice before being incubated for 3 h in medium supplemented with neutral red (50 μg/ml). The medium was washed off rapidly with a solution containing 0.5% formaldehyde and 1% calcium chloride. The cells were then incubated for a further 20 min at 37 $^0$C in a mixture of acetic acid (1%) and ethanol (50%) to extract the dye. The 96-well plate was then centrifuged at 2300×g for 5 min to settle down the debris if present in the solution. Following this 100 μl supernatant was transferred to new 96-well plate and the absorbance was measured at 540 nm using the microplate reader (Synergy-HT, BioTek).

***Quantitative real-time PCR analysis***

Cells were cultured in 6-well plates and exposed to CuO NPs (23 μg/ml) and/or curcumin (5 μg/ml) for 24 h. At the end of the exposure time, total RNA was extracted by

Qiagen RNeasy mini Kit (Valencia, CA, USA) according to the manufacturer's instructions. Concentration of the extracted RNA was determined using Nanodrop 8000 spectrophotometer (Thermo-Scientific, Wilmington, DE, USA), and the integrity of RNA was visualized on a 1% agarose gel using the gel documentation system (Universal Hood II, BioRad, Hercules, CA, USA). The first strand of cDNA was synthesized from 1 µg of total RNA by the reverse transcriptase using M-MLV (Promega, Madison, WI, USA) and oligo (dT) primers (Promega) according to the manufacturer's protocol. Quantitative real-time PCR was performed by QuantiTect SYBR Green PCR kit (Qiagen) using the ABI PRISM 7900HT Sequence Detection System (Applied Biosystems, Foster City, CA, USA). Two microliters of template cDNA were added to the final volume of 20 µl of reaction mixture. Real-time PCR cycle parameters included 10 minutes at 95°C followed by 40 cycles involving denaturation at 95°C for 15 seconds, annealing at 60°C for 20 seconds, and elongation at 72°C for 20 seconds. The sequences of the specific sets of primer for p53, bax, bcl-2, caspase-3 (Casp3), caspase-9 (Casp9) and β-actin used in this study are given in our previous publication (Ahamed et al., 2011). Expressions of selected genes were normalized to the β-actin gene, which was used as an internal housekeeping control.

***Mitochondrial membrane potential assay***

Red-orange cationic fluorescent dye tetramethylrhodamine, metyl ester, perchlorate (TMRM) (Thermo Fisher Scientific, Wyman Street, Waltham, MA, USA) is rapidly taken up by mitochondria in a potential-dependent manner. TMRM probe was applied to assess the mitochondrial membrane potential (MMP) level in control and treated cells. In brief, 20,000 cells/well seeded in a 96-well plate and allowed for 24 h to attach on the surface. Then, cells were treated for 24 h to CuO NPs (23 µg/ml) and/or curcumin (5 µg/ml). At the end of exposure time, cells were washed twice with PBS. Cells were further exposed with 100 nM of TMRM dye for 30 min at 37 °C in the dark. Cells were washed with PBS and fluorescent intensity of TMRM was quantified using a microplate reader (excitation/emission wavelength: 548/574 nm) (Synergy-HT, BioTek). A parallel set of experiment in 24-well plate ($1\times10^5$ cells/well) was also prepared as reported above. Then, intracellular brightness of TMRM was captured by a DMi8 fluorescent microscope (Leica Microsystems, GmbH, Wetzlar, Germany) using green excitation filter (detecting red-orange TMRM emission). Cell images were captured at 20× magnification.

***Reactive oxygen species (ROS) generation assay***

Intracellular ROS generation was measured using 2,7-dichlorofluorescin diacetate (DCFH-DA) as reported earlier (Siddiqui et al., 2013). ROS level was estimated through two procedures; quantitative analysis and microscopic fluorescence imaging. For quantitative assay, cells (20000 cells/well) were seeded in 96-well black-bottomed culture plates and allowed to adhere for 24 h in a $CO_2$ incubator at 37 °C. Further, cells were exposed for 24 h to CuO NPs (23 μg/ml) and/or curcumin (5 μg/ml). After the completion of exposure time, cells were washed twice with HBSS before being incubated in 1 ml of working solution of DCFH-DA at 37 °C for 30 min. Then, cells were lysed in alkaline solution and centrifuged at 2300×g for 15 min to settle down the cell debris. A 200 μl supernatant was transferred to a new 96-well plate, and fluorescence was measured at 485 nm excitation and 520 nm emission using a microplate reader (Synergy-HT, BioTek). The values were expressed as a percent of fluorescence intensity relative to the control cells. A parallel set of experiment in 24-well plate ($1\times10^5$ cells/well) was also prepared as reported above. Then, intracellular brightness of DCF probe (ROS level) was captured by a DMi8 fluorescent microscope (Leica Microsystems, GmbH, Wetzlar, Germany), with images taken at 20× magnification.

***Cell extract preparation***

Cell extrct was prepared for the measurements of malondialdehyde (MDA) level, glutathione (GSH) level, activity of apoptotic enzymes (caspase-3 and -9), along with several antioxidant enzymes such as glutathione peroxidase (GPx), superoxide dismutase (SOD), and catalase (CAT). Briefly, cells were cultured in 25-cm$^2$ culture flask and exposed for 24 h to CuO NPs (23 μg/ml) and/or curcumin (5 μg/ml). At the end of exposure time, cells were harvested in ice cold PBS by scraping and washed with PBS at 4 °C. Cell pellets were further lysed in cell lysis buffer [1X 20 mM Tris-HCl (pH 7.5), 150 mM NaCl, 1 mM $Na_2EDTA$, 1% Triton, 2.5 mM sodium pyrophosphate]. Following centrifugation (15000 g for 10 min at 4 °C) the supernatant (cell extract) was maintained on ice to perform the required experiments.

***Assay of caspase-3 and caspase-9 enzymes activity***

Colorimetric assay of caspase-3 and -9 enzymes activity was done using BioVision kits (Milpitas, CA, USA). This assay is based on the principle that activated caspases in apoptotic cells cleave the synthetic substrates to release free chromophore p-nitroanilide (pNA), which was recorded at 405 nm. The pNA produced after specific action of caspase-3 and caspase-9 on tertrapeptide substrates were DEVD-pNA and LEHD-pNA, respectively. In brief, reaction mixture consisted of 50 μl of control and treated cell extract protein (50 μg),

50 µl of 2× reaction buffer (containing 10 mM dithiothreitol) and 5 µl of 4 mM DEVD-pNA (for caspase- 3) or LEHD-pNA (for caspase-9) substrate in a total volume of 105 µl. The reaction mixture was incubated at 37 °C for1 h and absorbance of the product was measured using a microplate reader (Synergy-HT, BioTek) at 405 nm according to manufacturer's instruction.

***Malondialdehyde assay***

Malondialdehyde (MDA), an end product of lipid peroxidation was determined using methods of Ohkawa et al. (1979). Briefly, a mixture of 0.1 ml cell extract and 1.9 ml of 0.1 M sodium phosphate buffer (pH 7.4) was incubated at 37 °C for 1 h. Then, mixture was precipitated with 5% trichloroacetic acid (TCA) and centrifuged (2500×g) for 15 min to collect supernatant. Furthermore, 1.0 ml of 1% thiobarbituric acid (TBA) was added to the supernatant and placed in the boiling water for 15 min. After cooling to room temperature absorbance of the mixture was taken at 532 nm and was converted to MDA and expressed in nmole MDA/mg protein using molar extinction coefficient of $1.56 \times 10^5$ $M^-1$ $cm^-1$.

***Glutathione assay***

Glutathione (GSH) level was quantified using Ellman's method (Ellman, 1959). Briefly, a mixture of 0.1 ml of cell extract and 0.9 ml of 5% TCA was centrifuged (2300×g) for 15 min at 4 °C. Then, 0.5 ml of the supernatant was added into 1.5 ml of 0.01% 5,5'-dithiobis-(2-nitrobenzoic acid (DTNB) and the reaction was monitored at 412 nm. The amount of GSH was expressed in terms of nanomole/mg protein.

***Glutathione peroxidase enzyme assay***

Activity of glutathione peroxidase (GPx) enzyme was measured using protocol of Rotruck and co-workers (1973). In brief, a reaction mixture contained 20 µl of 0.1 M GSH, 10 µl of cell extract, 100 µl of 2 mM NADPH, 100 µl of 10 U/ml glutathione reductase, 800 µl of 0.2 M Tris-HCl, and 10 µl of 5 mM t-butylhydroperoxide. The oxidation rate of NADPH was monitored at 320 nm.

***Catalase enzyme assay***

Enzymatic activity of catalase was assayed according to the protocol of Sinha et al. (1972). In this method, dichromate in acetic acid is reduced to chromic acetate in the presence of $H_2O_2$. Briefly, reaction mixture (150 µl) contained 100 µl of 0.01M phosphate

buffer, 10 μl cell extract, and 40 μl of 2M $H_2O_2$ was prepared. The reaction was stopped by mixing of 200 μl of dichromoacetic acid reagent (5% of potassium dichromate and glacial acetic acid in 1:3 ratio) and absorbance was recorded at 530 nm.

***Superoxide dismutase enzyme assay***

Colorimetric assay of superoxide dismutase (SOD) enzyme was done using kit from Cayman Chemical Company (Michigan, OH, USA). https://www.caymanchem.com/product/706002/superoxide-dismutase-assay-kit

***Hydrogen peroxide assay***

Fluorometric assay of intracellular hydrogen peroxide ($H_2O_2$) was done using a commercial kit (MAK164 green fluorescence, Millipore-Sigma, St. Louis, MO, USA). https://www.sigmaaldrich.com/catalog/product/sigma/mak164?lang=en&region=US

## References


1. Ahamed M, Akhtar MJ, Siddiqui MA, et al (2011) Oxidative stress mediated apoptosis induced by nickel ferrite nanoparticles in cultured A549 cells. Toxicology 283:101–108. https://doi.org/10.1016/j.tox.2011.02.010
2. Ellman GL (1959) Tissue sulfhydryl groups. Archives of Biochemistry and Biophysics 82:70–77. https://doi.org/10.1016/0003-9861(59)90090-6
3. Mosmann T (1983) Rapid colorimetric assay for cellular growth and survival: Application to proliferation and cytotoxicity assays. Journal of Immunological Methods 65:55–63. https://doi.org/10.1016/0022-1759(83)90303-4
4. Ohkawa H, Ohishi N, Yagi K (1979) Assay for lipid peroxides in animal tissues by thiobarbituric acid reaction. Analytical Biochemistry 95:351–358. https://doi.org/10.1016/0003-2697(79)90738-3
5. Rotruck JT, Pope AL, Ganther HE, et al (1973) Selenium: Biochemical role as a component of glatathione peroxidase. Science 179:588–590. https://doi.org/10.1126/science.179.4073.588
6. Siddiqui MA, Alhadlaq HA, Ahmad J, et al (2013) Copper Oxide Nanoparticles Induced Mitochondria Mediated Apoptosis in Human Hepatocarcinoma Cells. PLoS ONE 8:. https://doi.org/10.1371/journal.pone.0069534
7. Sinha AK (1972) Colorimetric assay of catalase. Analytical Biochemistry 47:389–394.

https://doi.org/10.1016/0003-2697(72)90132-7
